# The Effects of Selective Serotonin Reuptake Inhibitors on Neurological and Depressive Symptoms in Multiple Sclerosis: A Systematic Review and Meta-analysis of Randomized Controlled Trials

**DOI:** 10.31661/gmj.v12i.3153

**Published:** 2023-12-18

**Authors:** Faeze Yousefi, Parnia Kamyab, Bahareh Fakhraei, Mojtaba Farjam, Shahla Rezaei, Seyed Sasan Mahmoudi, Zeinab Karimimoghadam, Reza Tabrizi, Nematollah Jaafari

**Affiliations:** ^1^ Student Research Committee, Fasa University of Medical Sciences, Fasa, Iran; ^2^ Research Center for Psychiatry and Behavioral Sciences, Shiraz University of Medical Sciences, Shiraz, Iran; ^3^ Department of Psychiatry, Fasa University of Medical Sciences, Fasa, Iran; ^4^ Clinical Research Development Unit, Valiasr Hospital, Fasa University of Medical Sciences, Fasa, Iran; ^5^ Noncommunicable Diseases Research Center, Fasa University of Medical Sciences, Fasa, Iran; ^6^ Nutrition Research Center, School of Nutrition and Food Sciences, Shiraz University of Medical Sciences, Shiraz, Iran; ^7^ Student Research Committee, Department of Neurosurgery, School of Medicine, Shiraz University of Medical Sciences, Shiraz, Iran; ^8^ University of Poitiers, Center for Research on Cognition and Learning CNRS 7295, Clinical Research Unit in Psychiatry of the Center Hospitalier Henri Laborit 86000, Poitiers, France

**Keywords:** Selective Serotonin Reuptake Inhibitors, Multiple Sclerosis, Randomized Controlled Trial

## Abstract

Background: Multiple sclerosis (MS) affects the central nervous system and creates plaques by demyelination of neurons. Several studies have investigated the effect of selective serotonin reuptake inhibitors (SSRIs) on MS clinical courses. The current meta-analysis was conducted to determine the effect of SSRIs on neurological and depressive symptoms of MS disease based on a systematic review and meta-analysis of randomized controlled trials.Materials and Methods: We searched the PubMed/Medline, Scopus, EMBASE, Google scholar, Web of Science, and Cochrane Library until June 2023. The effects of SSRI were assessed through indictors such as symbol digit modalities test (SDMT), expanded disability status scale (EDSS), modified fatigue impact scale (MFIS), and Beck’s depression inventory/psychiatric (BDI).Results: Considering the inclusion criteria, seven articles (including eight trials) were included in this review. The meta-analysis results demonstrated that SSRIs treatments did not have significant effects on indicators of neurological and depressive symptoms, such as SDMT (Weighted Mean Difference (WMD)=-0.87; 95% CI, -7.74, 5.99, P=0.35; I2=0.0%), EDSS (WMD=-0.05; 95% CI, -0.24, 0.14, P=0.62; I2=0.0%), MFIS (WMD=5.29; 95% CI, -18.10, 28.68, P=0.21; I2=0.0%), and BDI (WMD=-0.47; 95% CI, -2.61, 1.67, P=0.67; I2=32.05%) in patients with MS compared with controls. Conclusion: This study shows that the consumption of SSRIs in MS patients compared to the control group does not bring about a significant change in the indices related to neurological and depressive symptoms. Further meta-analyses are required in order to provide stronger evidence in the future.

## Introduction

Multiple sclerosis (MS) is a chronic immune-mediated central nervous system disorder identified by demyelination and degeneration of axons [1]. The signs and symptoms of MS vary widely and depend on the affected nerves and their extent of damage [2]. The pathophysiology and underlying mechanisms of this disease are complex and still unclear, although some articles focus on the role of infection and genetics, as well as immune-mediated mechanisms [3][4][5].

MS is associated with neurological disorders, especially among youth, and can bring about some presentations such as weakness and lethargy, vision problems, fatigue, and bladder dysfunction [6]. Based on the severity and type of attacks, MS occurs in four phenotypes including clinically isolated syndrome, remitting-relapsing, primary progressive, and secondary progressive multiple sclerosis [7].

MS is more common in women than men, and according to the latest statistics published in 2020, it is estimated that it has affected nearly 3 million people in the world [8]. A recent systematic review and meta-analysis by Mirmossayeb et al has declared the pooled prevalence of MS 100 in 100,000 among Iranian population [9]. Currently, there is no definitive treatment for this disease, although some drugs can modify the clinical manifestations in the early stages [10]. In addition to neurologic courses of MS, patients may experience a range of psychiatric symptoms such as depression and less commonly, anxiety during attacks [11].

Some believe that depression and MS have a double effect on each other, so that MS attacks cause depression and depression aggravates MS attacks [12]. According to a systematic review by Ann Marrie et al, the prevalence of depression among the MS population was reported to be 23.7% [13].

In this regard, multiple studies have investigated the effect of antidepressants in MS patients from different aspects. For instance, Fluoxetine, a selective serotonin reuptake inhibitor commonly used in psychiatric disorders, is recognized to probably have neuroprotective effects resulting in a reduction in inflammatory reactions in MS patients [14][15]. Meanwhile, according to the randomized clinical trial by Mostert et al, fluoxetine was not beneficial in the progression of MS compared to placebo [16]. Sertraline was another drug that was investigated from the selective serotonin reuptake inhibitor (SSRI) family. In accordance with the study conducted by Mohr et al, Sertraline was effective in reducing at least 50% of depression symptoms in MS patients [17].

As mentioned above, there are still conflicting results regarding the effect of SSRIs among MS patients. This study was conducted with the aim of investigating the available evidence regarding the effect of SSRI drugs on neurological and depressive symptoms in MS patients using a systematic review and meta-analysis method.

## Materials and Methods

The present systematic review and meta-analysis were conducted and reported based on the Preferred Reporting Items for Systematic Reviews and Meta-Analyses (PRISMA) checklist with PROSPERO registration number: CRD42021234585 (available at: https://www.crd.york.ac.uk/prospero/#myprospero).

Electronic searches were systematically performed in PubMed/Medline, Scopus, EMBASE, Google scholar, Web of Science, and Cochrane Library until June 2023 to identify all randomized controlled trials (RCTs) investigating the effects of SSRIs in MS. The following MeSH terms and keywords were used: "SSRIs", "sertraline", "paroxetine", "citalopram", "escitalopram", "fluvoxamine", "neurologic symptoms", "depressive symptoms", " MS", and "RCT". The search strategy of the PubMed database is included as supplemental information (Suppl File-1). Additionally, the reference lists of previous reviews and included articles were checked to identify further studies.

Study Selection

Two independent authors (FY and PK) checked the titles and abstracts to identify related articles and remove irrelevant and duplicate reports. Subsequently, full papers of the remaining articles were retrieved for further assessment of their eligibility for the current meta-analysis. Articles were selected for the meta-analysis based on the following inclusion criteria: being an RCT, conducted on human subjects with MS, and reporting mean changes between before and after treatment with standard deviations (SDs) or corresponding 95% confidence intervals (CIs) on indicators of neurological and depressive symptoms, such as symbol digit modalities test (SDMT), expanded disability status scale (EDSS), modified fatigue impact scale (MFIS), and Beck’s depression inventory/psychiatric ( ) following SSRIs intake for the treatment and control groups. Other types of studies, investigations without a comparison group, and studies without sufficient data were excluded.

Data Extraction and Quality Assessment

Two authors (PK and FY) independently extracted all relevant data using a standardized Excel file and a third author cross-checked for accuracy. The extracted data included the first author’s name, year of publication, number of participants in intervention/control groups, main characteristics of participants, medication types, dosage and duration of treatment, control types, and the mean and standard deviation (SD) for SDMT, EDSS, MFIS, and BDI in the treatment and control groups. SDMT, EDSS, and MFIS serve as indicators for neurological symptoms, while BDI is used as an indicator for depressive symptoms. Data extraction was performed only for indicators with at least two studies. Risk of Bias (RoB) assessment of included trials utilized the Cochrane Collaboration risk of bias tool, considering randomization generation, allocation concealment, blinding of participants and outcome assessment, incomplete outcome data, selective outcome reporting, and other potential sources of bias. Disagreements were resolved through consensus and discussion.

Statistical Analysis

We assessed the effects of SSRI intake on changes in the studied indicators, including: 1) SDMT, 2) EDSS, 3) MFIS, and 4) BDI. Pooled effect sizes, along with weighted mean differences (WMDs) and 95% CIs, were determined using a random-effects model in the current meta-analysis. Inter-study heterogeneity was evaluated using Cochran’s Q and I-square tests, with significant heterogeneity defined as a Cochran’s Q test P-value<0.1 and I-square>50%. For outcomes with less than two studies, standard-error adjustment using the Knapp-Hartung method was applied. All statistical analyses were conducted using STATA software version 12.0 (Stata Corp., College Station, TX) and RevMan V.5.3 software (Cochrane Collaboration, Oxford, UK).

## Results

**Figure-1:**
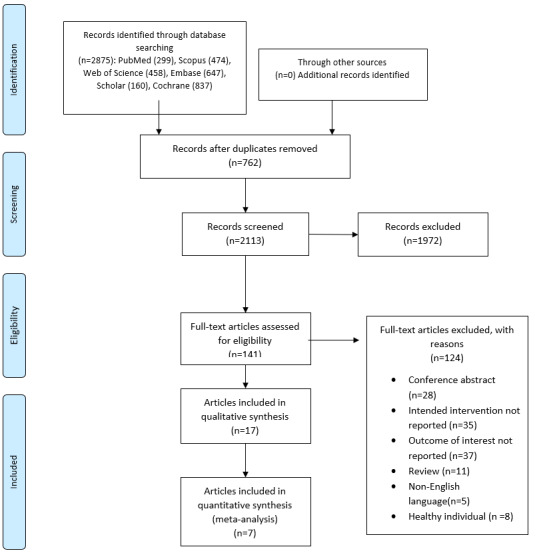
Figure 1. PRISMA flowchart of study identification and Selection

**Figure-2:**
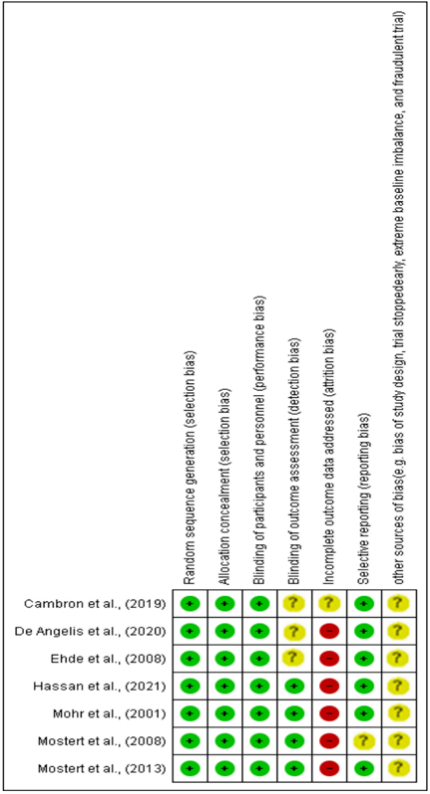
Figure 2. Quality assessment results using the Cochrane RoB tool

Figure-1 depicts the study identification and selection process. Finally, seven articles [12][15][17][18][19][20][21] (including eight trials) out of 2875 citations were found suitable for the current meta-analysis. Due to different control groups in study conducted by Mohr et al. [17] we included it as two separate studies. Among these, four trials investigated the effects of SSRIs on BDI, three on EDSS, and two on SDMT and MFIS. In total, 562 MS patients were randomly assigned (278 patients in the treatment group and 284 in the comparison group) in the current meta-analysis. The duration of treatment varied from 6 weeks to 108 weeks. The included articles were published between January 2001 and June 2023. The main characteristics of the included trials are presented in Table-1. The quality assessment findings, as judged by the authors, are shown in Figure-2.

Main Outcomes

**Figure-3:**
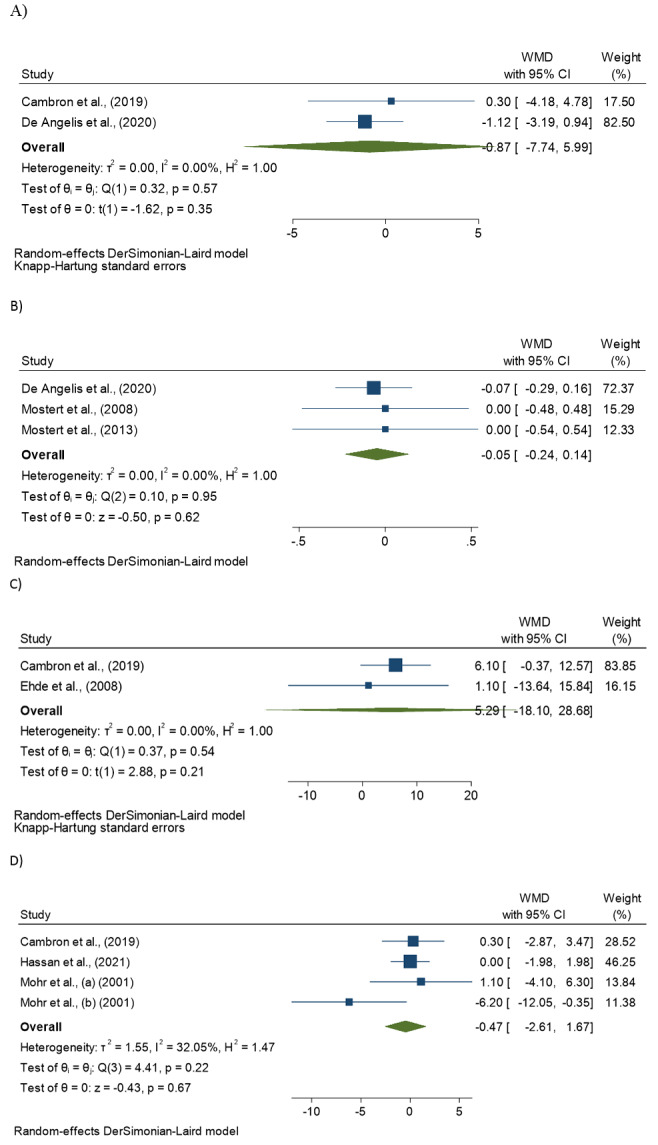
Figure 3. Forest plots indicting the effects of SSRIs on SMDT (A), EDSS (B), MFIS (C), BDI (D) in patients with MS

Forest plots illustrating the effects of SSRIs on indicators of neurological and depressive symptoms are shown in Figure-3. The meta-analysis results using a random-effects model demonstrated that SSRIs treatments did not have significant effects on indicators of neurological and depressive symptoms, such as SDMT (WMD=-0.87; 95% CI, -7.74, 5.99, P=0.35; I2=0.0%), EDSS (WMD=-0.05; 95% CI, -0.24, 0.14, P=0.62; I2=0.0%), MFIS (WMD=5.29; 95% CI, -18.10, 28.68, P=0.21; I2=0.0%), and BDI (WMD=-0.47; 95% CI, -2.61, 1.67, P=0.67; I2=32.05%) in patients with MS compared with controls.

Sensitivity Analysis

**Figure-4:**
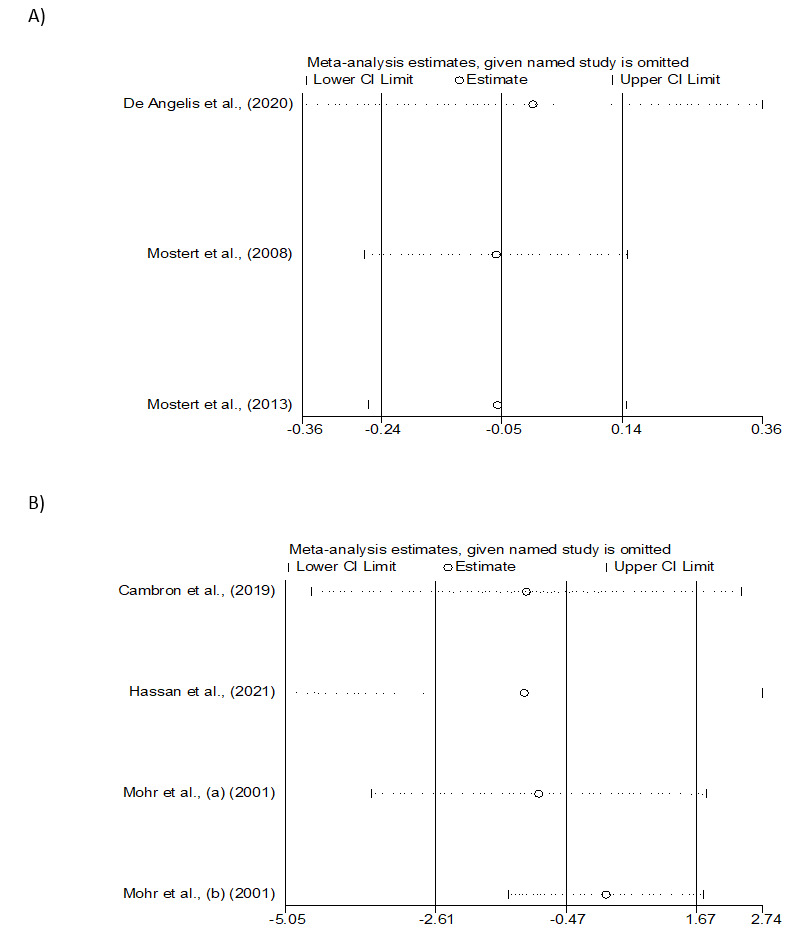
Figure 4. Sensitivity results for EDSS (A) and BDI (B).

In sensitivity analysis, we found no significant changes between pre- and post-sensitivity analysis for EDSS and BDI indicators. However, the lower and higher pooled WMDs in sensitivity analyses of EDSS and BDI are shown in Figure-4. Unfortunately, additional analyses, such as subgroup and meta-regression, and publication bias analyses were not possible due to the limited number of included studies.

## Discussion

**Table:** Table1. Main Characteristics of Included Studies

**Authors**	**Country**	**Population**	**Gender**	**Medications/ control group **	**Dosage of intervention**	**Duration of intervention**	**Mean age** **(intervention/ control groups ) **
Cambron et al., (2019) [21][A1]	Belgium	PPMS/SPMS	F	Fluoxetine/ Placebo	20 mg/day	108 weeks	54±6.11, 51.2±7.64
De Angelis et al., (2020) [20]	UK	SPMS	M/F	Fluoxetine/ Placebo	20 mg/day	96 weeks	54.83±7.1, 54.89±7.16
Ehde et al., (2008) [19]	USA	MS and MDD	M/F	Paroxetine/ Placebo	10 mg/day for 1 week and 20-40 mg/day after 1 week	12 weeks	≥18 years old
Hassan et al., (2021) [12]	Egypt	RRMS	M/F	SSRI/ rTMS	NR	6 weeks	31.2±4.7, 29.3±3.7
Mohr et al., (a) (2001) [17]	USA	MS and MDD	M/F	Sertraline/ CBT	88.75 mg/day	16 weeks	43.9±10, 43.9±10
Mohr et al., (b) (2001) [17]	USA	MS and MDD	M/F	Sertraline/ SEG	88.75 mg/day	16 weeks	43.9±10, 43.9±10
Mostert et al., (2008) [15]	Netherlands	RRMS	M/F	Fluoxetine/ Placebo	20 mg/day	24weeks	41±10, 38±9
Mostert et al., (2013) [18]	Netherlands	PPMS/SPMS	M/F	Fluoxetine/ Placebo	40 mg/day	104 weeks	49.7±9.2, 47.5±7.6

^*^Abbreviation: **F:** Female; **M:** male; **PPMS:** primary progressive multiple sclerosis; **SPMS:** secondary progressive multiple sclerosis; **MS:** multiple sclerosis; **MDD:** major depressive disorder; **RRMS:** relapsing-remitting multiple sclerosis; **SSRIs:** selective serotonin reuptake inhibitors; **rTMS:** repetitive transcranial magnetic stimulation; **CBT:** cognitive behavioral therapy; **SEG:** supportive-expressive group therapy; **NR:** not reporting.

This comprehensive systematic review and meta-analysis was conducted in order to evaluate the effect of SSRIs on neurological and depressive symptoms in patients with multiple sclerosis based on randomized clinical trials. Previous studies have reported higher rates of depression and anxiety as well as the use of antidepressants among MS patients [22]. As a matter of fact, depression could be as a result of awareness of a progressive and debilitating disease [23]. In addition, MS patients are more susceptible for depression through a variety of socio-psychological risk factor like inefficiency in everyday tasks and lack of social support [24]. Moreover, according to available resources, depression can aggravate symptoms in MS patients through its effect on immune mechanisms or reducing adherence to treatment [25][26]. It has also been revealed that depression in this group of patients is associated with a decrease in the quality of life and an enhanced chance of suicide [24][27]. These are all things that bring us to the importance of treating depression in MS patients.

Based on the results, SSRI treatments did not significantly affect either the neurological symptoms indicators (SDMT, EDSS, and MFIS) or the single indicator of depressive symptoms (BDI). This conclusion was assessed through SDMT, EDSS, MFIS, and BDI. In line with our study, according to a meta-analysis in 2014, SSRIs were evaluated for their disease-modifying effects in multiple sclerosis patients and results indicated no significant improvement in major depressive disorder between SSRI-treated and control groups [28]. In addition to that, the effect of drugs on the level of fatigue and quality of life of these patients was also investigated, and no significant relationship was observed, and even cases of increased headache and nausea were reported [28]. In this project, we tried to measure the effect of SSRI drugs in MS patients in terms of various indicators, including depressive symptom, fatigue, neurocognitive function, and disability progression. However, according to a study on a mouse model of multiple sclerosis by Bhat et al, fluoxetine delayed the onset of the disease and also reduced the rate of neuro-inflammatory reactions [14]. Although this research had significant results, it was not included in our meta-analysis due to its non-human subjects. The absence of a significant association between SSRI consumption and indicators of neurological attacks and depressive symptoms in MS patients might be attributed to the limited pool of available studies which included a constrained population. Consequently, while not statistically significant, this finding holds valuable implications, underscoring the importance of acknowledging the limitations and complexities present in the available dataset. The mechanism considered to explain was that fluoxetine suppresses the immune response in experimental autoimmune encephalomyelitis by reducing the secretion of cytokines [14]. Moreover, according to the previous investigations, it has been demonstrated that Serotonin (5-hydroxytryptophan) plays an important role in modulating reactions of the immune system through activating T cells and natural killer cells and as a result of that, a selective serotonin reuptake inhibitor it can reduce the severity of symptoms in MS patients by modulating immune and inflammatory reactions [29]. The current study does offer valuable insights, yet it does have some limitations worth acknowledging. First, we focused solely on human studies, which means we missed insights from research on non-human models. Additionally, the limited number of eligible studies in our final analysis means that we need to interpret the results cautiously. Therefore, it’s important to conduct up-to-date meta-analyses to include a broader range of original articles in the future, which would help improve the overall depth and reliability of findings.

## Conclusion

In conclusion, the present study explored the impact of SSRIs on neurological symptoms and psychological facets in patients with MS. Our findings suggest that SSRIs do not significantly alter considered indicators including SDMT, EDSS, MFIS, and BDI. This study provides valuable insights into the limited influence of SSRIs on MS-related attacks. Further research is essential for a comprehensive understanding and potential advancements in treatment strategies for MS patients.

## Acknowledgment

This article received approval from the Ethics Committee of Fasa Medical University with code IR.FUMS.REC.1400.146 and was supported by The Deputy of Research and Technology of Fasa University of Medical Sciences, Fasa, Iran, with grant number 400160.

## Conflict of Interest

The authors declare no conflict of interest.
